# Risk of Death Associated With Reversion From Prediabetes to Normoglycemia and the Role of Modifiable Risk Factors

**DOI:** 10.1001/jamanetworkopen.2023.4989

**Published:** 2023-03-28

**Authors:** Zhi Cao, Wenyuan Li, Chi Pang Wen, Shu Li, Chen Chen, Qingqing Jia, Wanlu Li, Weiqi Zhang, Huakang Tu, Xifeng Wu

**Affiliations:** 1 Center for Clinical Big Data and Analytics, The Second Affiliated Hospital and School of Public Health, Zhejiang University School of Medicine, Hangzhou, Zhejiang, China; 2 Alibaba-Zhejiang University Joint Research Center of Future Digital Healthcare, Hangzhou, Zhejiang, China; 3 Key Laboratory of Intelligent Preventive Medicine of Zhejiang Province, Hangzhou, Zhejiang, China; 4 National Institute for Data Science in Health and Medicine, Zhejiang University, Hangzhou, Zhejiang, China; 5 School of Medicine and Health Sciences, George Washington University, Washington, District of Columbia; 6 Cancer Center, Zhejiang University, Hangzhou, Zhejiang, China

## Abstract

**Importance:**

Individuals with prediabetes have a higher risk of death than healthy individuals. However, previous findings have suggested that individuals with reversion from prediabetes to normoglycemia may not have a lower risk of death compared with individuals with persistent prediabetes.

**Objectives:**

To investigate the associations between changes in prediabetes status and risk of death and to elucidate the roles of modifiable risk factors in these associations.

**Design, Setting, and Participants:**

This population-based prospective cohort study used data from 45 782 participants with prediabetes from the Taiwan MJ Cohort Study who were recruited between January 1, 1996, and December 31, 2007. Participants were followed up from the second clinical visit to December 31, 2011, with a median (IQR) follow-up of 8 (5-12) years. Participants were categorized into 3 groups according to changes in their prediabetes status within a 3-year period after initial enrollment: reversion to normoglycemia, persistent prediabetes, and progression to diabetes. Cox proportional hazards regression models were used to examine the associations between changes in prediabetes status at baseline (ie, the second clinical visit) and risk of death. Data analysis was performed between September 18, 2021, and October 24, 2022.

**Main Outcomes and Measures:**

All-cause mortality, cardiovascular disease (CVD)–related mortality, and cancer-related mortality.

**Results:**

Of 45 782 participants with prediabetes (62.9% male; 100% Asian; mean [SD] age, 44.6 [12.8] years), 1786 (3.9%) developed diabetes and 17 021 (37.2%) reverted to normoglycemia. Progression from prediabetes to diabetes within a 3-year period was associated with higher risks of all-cause death (hazard ratio [HR], 1.50; 95% CI, 1.25-1.79) and CVD-related death (HR, 1.61; 95% CI, 1.12-2.33) compared with persistent prediabetes, while reversion to normoglycemia was not associated with a lower risk of all-cause death (HR, 0.99; 95% CI, 0.88-1.10), cancer-related death (HR, 0.91; 95% CI, 0.77-1.08), or CVD-related death (HR, 0.97; 95% CI, 0.75-1.25). Among individuals who were physically active, reversion to normoglycemia was associated with a lower risk of all-cause death (HR, 0.72; 95% CI, 0.59-0.87) compared with those with persistent prediabetes who were physically inactive. Among individuals with obesity, risk of death varied between those who experienced reversion to normoglycemia (HR, 1.10; 95% CI, 0.82-1.49) and those who had persistent prediabetes (HR, 1.33; 95% CI, 1.10-1.62).

**Conclusions and Relevance:**

In this cohort study, although reversion from prediabetes to normoglycemia within a 3-year period did not mitigate the overall risk of death compared with persistent prediabetes, risk of death associated with reversion to normoglycemia varied based on whether individuals were physically active or had obesity. These findings highlight the importance of lifestyle modification among those with prediabetes status.

## Key Points

**Question:**

Is reversion from prediabetes to normoglycemia associated with lower risk of death?

**Findings:**

In this cohort study of 45 782 participants with prediabetes, reversion to normoglycemia within a 3-year period was not associated with a lower overall risk of death compared with individuals with persistent prediabetes. Reversion to normoglycemia was associated with a lower risk of all-cause death among those who were physically active; among individuals with obesity, risk of death varied between those who experienced reversion to normoglycemia and those who had persistent prediabetes.

**Meaning:**

These findings support the importance of lifestyle modification among people with prediabetes and provide new evidence to identify targeted interventions that may produce greater benefits for this population.

## Teaser

This cohort study examines the associations between changes in prediabetes status and risk of death among Asian individuals with prediabetes and explores the roles of modifiable risk factors in these associations.

## Introduction

Prediabetes, defined by glycemic indicators that are higher than normal but lower than diabetes thresholds, is considered a high-risk state for type 2 diabetes.^1^ Globally, 352 million people are living with prediabetes, and the number is expected to increase to 587 million by the year 2045.^2^ The latest nationally representative survey in China revealed that the estimated prevalence of prediabetes was 38.1% in 2018.^3,4^ Prediabetes status is commonly characterized by mild insulin resistance and β-cell dysfunction.^1^ Like type 2 diabetes, prediabetes has been suggested to be associated with higher risks of cardiovascular disease (CVD), chronic kidney disease, cancer, dementia, and death.^5^

Approximately 25% of individuals with prediabetes will develop diabetes within 3 to 5 years, and as many as 70% of individuals with prediabetes will progress to diabetes in their lifetime.^1,6^ Progression from prediabetes to diabetes has been associated with a higher risk of death.^7,8,9,10^ Nevertheless, evidence of the association between reversion from prediabetes to normoglycemia and the risk of death remains inconsistent.^8,9,10,11^ The discrepancy between the findings of previous studies^7,8,9,10,11^ may be due to different study populations and sample sizes. In addition, there is a lack of evidence regarding the associations between changes in prediabetes status and cause-specific mortality.^10^ Furthermore, few studies have examined the roles of modifiable risk factors in the associations between changes in prediabetes status and risk of death. In the present study, we aimed to examine the associations between changes in prediabetes status within 1 to 3 years and the long-term risks of all-cause death, cancer-related death, and CVD-related death and to explore the roles of modifiable risk factors in death risk stratification among those with reversion to normoglycemia in a large prospective cohort.

## Methods

### Study Design and Population

The study was based on data from the prospective Taiwan MJ cohort study,^12,13,14,15^ which enrolled more than 500 000 individuals undergoing a standardized comprehensive medical screening program across Taiwan since 1996. At enrollment, every participant completed a self-administered questionnaire on demographic information, lifestyle, and medical history and underwent a series of anthropometric measurements, physical examinations, and blood and urinary tests during clinical visits. Details of the Taiwan MJ cohort and data collection have been reported elsewhere.^12,13,14,15^ The institutional review boards of the MJ Health Management Institution and the National Health Research Institutes approved this study. Written informed consent authorizing data processing was obtained from all participants. This study followed the Strengthening the Reporting of Observational Studies in Epidemiology (STROBE) reporting guideline for cohort studies.

Overall, we identified 157 866 adults with prediabetes status at their first clinical visit between January 1, 1996, and December 31, 2007. We then excluded individuals who had an interval of less than 1 year or more than 3 years between the first 2 clinical visits, had missing data on fasting plasma glucose (FPG) level in the second clinical visit, had a history of cancer, or died within the first year after enrollment. Those who had type 1 diabetes or gestational diabetes at the first clinical visit were excluded. A total of 45 782 individuals were eligible for inclusion in the present analysis (eFigure 1 in Supplement 1). Participants were followed up from the second clinical visit to December 31, 2011. Data were analyzed between September 18, 2021, and October 24, 2022.

### Ascertainment of Prediabetes, Diabetes, and Normoglycemia

An overnight fasting blood sample was taken in the morning, and plasma glucose concentrations were measured using an automatic biochemical analyzer (7150; Hitachi). The classifications of prediabetes, diabetes, and normoglycemia were based on American Diabetes Association criteria.^16^ Prediabetes was defined as an FPG level of 100 mg/dL to 125 mg/dL (to convert glucose levels to millimoles per liter, multiply by 0.0555). Diabetes was defined as an FPG level of 126 mg/dL or higher, a physician’s diagnosis of diabetes, and/or the use of antidiabetic medications. Normoglycemia was defined as an FPG level of less than 100 mg/dL. Participants were grouped according to changes in their prediabetes status within 1 to 3 years, which included progression to diabetes, persistent prediabetes, or reversion to normoglycemia.

### Follow-up for Mortality

The outcomes of interest were all-cause mortality, cancer-related mortality, and CVD-related mortality. The mortality data were obtained through the Taiwan death file,^12^ which records the date of death and the underlying cause of death using codes from the *International Classification of Diseases, Ninth Revision* (*ICD-9*), and the *International Statistical Classification of Diseases and Related Health Problems, Tenth Revision* (*ICD-10*). These codes were used to identify cancer-related mortality (*ICD-9* codes 140-239 and *ICD-10* codes C00-C97) and CVD-related mortality (*ICD-9* codes 390-459 and *ICD-10* codes I00-I99). Participants were followed up starting at the second clinical visit until the date of death or the end of the follow-up period (December 31, 2011), whichever came first.

### Covariate Assessment

A standard self-administered questionnaire was used to collect the following information at the second clinical visit: sociodemographic factors (including age, sex, educational attainment, occupation, and marital status), lifestyle factors (including smoking status, alcohol drinking status, body mass index [BMI] [calculated as weight in kilograms divided by height in meters squared], physical activity level, and sugar-sweetened beverage intake), and medical history (including hypertension and total cholesterol level). Seated blood pressure was measured using an autosphygmomanometer (CH-5000; Citizen). Hypertension was defined as systolic blood pressure of 140 mm Hg or higher, diastolic blood pressure of 90 mm Hg or higher, or the use of antihypertensive medications. Total cholesterol concentrations were measured using the same automatic biochemical analyzer.

We considered physical activity level, smoking status, alcohol drinking status, BMI, and dietary patterns (intake of vegetables, fruits, and sugar-sweetened beverages) as modifiable risk factors, which were measured at baseline (ie, the second clinical visit). Participants were asked to report the intensity, frequency, and duration of physical activity during the previous month; they were then divided into 3 categories: inactive (<3.75 metabolic equivalent of task [MET] hours per week), moderately active (3.75-7.49 MET hours per week), or active (≥7.50 MET hours per week).^13,17^ We defined normal weight as a BMI of less than 24.0, overweight as a BMI of 24.0 to 27.8, and obesity as a BMI of 28.0 or higher. Data on smoking status (never, former, or current), alcohol drinking status (never, former, or current), vegetable intake (<1 serving per day, 1-2 servings per day, or >2 servings per day), fruit intake (<1 serving per day, 1-2 servings per day, or >2 servings per day), and sugar-sweetened beverage intake (0 mL per week, 240-720 mL per week, or >720 mL per week) were collected through the self-administered questionnaire.

### Statistical Analysis

Baseline characteristics were reported as means with SDs for continuous variables and as numbers with percentages for categorical variables. A χ^2^ test was used for categorical variables, and analysis of variance was used for continuous variables.

Cox proportional hazard models were used to estimate hazard ratios (HRs) and 95% CIs for the associations between changes in prediabetes status within a 3-year period and the risks of all-cause death, cancer-related death, and CVD-related death, with persistent prediabetes as the reference group. The proportional hazards assumption was checked by tests based on Schoenfeld residuals, and the results suggested that the assumptions were not violated. The percentages of missingness for covariates were relatively low (≤7.8%), and multiple imputation by chained equations with 10 imputations was performed.

To assess the roles of modifiable risk factors in the change of glucose, multinomial logistic regression models were used to estimate odds ratios (ORs) and 95% CIs for the associations of modifiable risk factors with changes in prediabetes status after adjusting for covariates. Tests for the interactions between changes in prediabetes status and modifiable risk factors were performed by fitting an interaction term between the exposure of interest and each risk factor. Moreover, to quantify the gain or loss of life-years between persistent prediabetes and reversion to normoglycemia across modifiable risk factors, differences in life expectancy were estimated using flexible parametric survival models.^18^

A series of sensitivity analyses were performed to evaluate the robustness of our findings. First, to assess whether the risk of death varied across the definitions of prediabetes, we defined prediabetes status as an FPG level of 110 mg/dL to 125 mg/dL according to World Health Organization criteria.^19^ Second, antihypertensive and lipid-lowering medications that might be used among those with prediabetes were further adjusted in the multivariable model. Third, to account for competing events, we used the Fine-Gray competing risk model for the analysis of cause-specific risk of death. Fourth, we conducted a landmark analysis that excluded deaths occurring within 2 years after the second clinical visit to minimize the potential contribution of reverse causality to these findings. Fifth, the main analyses were repeated by restricting the analyses to participants with complete data on all covariates. Sixth, E-values (risk ratios) were calculated to assess the effects of unmeasured confounding.^20^

All analyses were performed using Stata statistical software, version 13 (StataCorp LLC). All tests were 2-sided, and *P* < .05 was considered statistically significant.

## Results

### Baseline Characteristics of Population

Of 45 782 participants with prediabetes (28 770 male [62.9%] and 17 003 female [37.1%]; 100% Asian; mean [SD] age, 44.6 [12.8] years), 1786 (3.9%) developed type 2 diabetes and 17 021 (37.2%) reverted to normoglycemia within 3 years after enrollment. During a median (IQR) follow-up of 8 (5-12) years, 1528 deaths occurred, including 671 from cancer and 308 from CVD. Population characteristics by change in prediabetes status are shown in Table 1.

**Table 1.** Baseline Characteristics of Participants by Changes in Prediabetes Status

Characteristic	Participants, No./total No. (%)				*P* value
	Total (N = 45 782)	Change in prediabetes status			
		Reversion to normoglycemia (n = 17 021)	Persistent prediabetes (n = 26 975)	Progression to diabetes (n = 1786)	
Sex					
Male	28 779/45 782 (62.9)	9858/17 021 (57.9)	17 855/26 975 (66.2)	1066/1786 (59.7)	<.001
Female	17 003/45 782 (37.1)	7163/17 021 (42.1)	9120/26 975 (33.8)	720/1786 (40.3)	
Age, mean (SD), y	44.6 (12.8)	42.0 (12.6)	45.8 (12.6)	51.3 (12.3)	<.001
Marital status					
Single	6696/43 420 (15.4)	3200/16 188 (19.8)	3386/25 544 (13.3)	110/1688 (6.5)	<.001
Married or cohabiting	33 603/43 420 (77.4)	11 941/16 188 (73.8)	20 255/25 544 (79.3)	1407/1688 (83.4)	
Divorced or widowed	3121/43 420 (7.2)	1047/16 188 (6.5)	1903/25 544 (7.4)	171/1688 (10.1)	
Educational attainment					
Middle school or lower	8996/44 143 (20.4)	2852/16 428 (17.4)	5528/25 998 (21.3)	616/1717 (35.9)	<.001
High school	21 731/44 143 (49.2)	8406/16 428 (51.2)	12 550/25 998 (48.3)	775/1717 (45.1)	
College or higher	13 416/44 143 (30.4)	5170/16 428 (31.5)	7920/25 998 (30.5)	326/1717 (19.0)	
Occupation					
Blue collar	17 431/43 169 (40.4)	6354/16 113 (39.4)	10 337/25 392 (40.7)	740/1664 (44.5)	<.001
Self-employed	13 171/43 169 (30.5)	5008/16 113 (31.1)	7683/25 392 (30.3)	480/1664 (28.8)	
Student	644/43 169 (1.5)	316/16 113 (2.0)	311/25 392 (1.2)	17/1664 (1.0)	
White collar	5231/43 169 (12.1)	1899/16 113 (11.8)	3176/25 392 (12.5)	156/1664 (9.4)	
Other^a^	6692/43 169 (15.5)	2536/16 113 (15.7)	3885/25 392 (15.3)	271/1664 (16.3)	
Smoking status					
Never	28 286/42 558 (66.5)	10 727/15 774 (68.0)	16 526/25 136 (65.7)	1033/1648 (62.7)	<.001
Former	3806/42 558 (8.9)	1241/15 774 (7.9)	2409/25 136 (9.6)	156/1648 (9.5)	
Current	10 466/42 558 (24.6)	3806/15 774 (24.1)	6201/25 136 (24.7)	459/1648 (27.8)	
Alcohol drinking status					
Never	31 108/42 221 (73.7)	11 905/15 680 (75.9)	18 047/24 904 (72.5)	1156/1637 (70.6)	<.001
Former	1286/42 221 (3.0)	429/15 680 (2.7)	790/24 904 (3.2)	67/1637 (4.1)	
Current	9827/42 221 (23.3)	3346/15 680 (21.3)	6067/24 904 (24.4)	414/1637 (25.3)	
Sugar-sweetened beverage intake, mL/wk					
0	17 374/45 782 (37.9)	6403/17 021 (37.6)	10 188/26 975 (37.8)	783/1786 (43.8)	<.001
240-720	12 028/45 782 (26.3)	4430/17 021 (26.0)	7173/26 975 (26.6)	425/1786 (23.8)	
>720	16 380/45 782 (35.8)	6188/17 021 (36.4)	9614/26 975 (35.6)	578/1786 (32.4)	
Hypertension	11 700/45 782 (25.6)	3153/17 021 (18.5)	7699/26 975 (28.5)	848/1786 (47.5)	<.001
Physical activity, mean (SD), MET h/wk	6.2 (8.7)	5.9 (8.4)	6.4 (8.8)	6.2 (9.1)	<.001
Fruit intake, mean (SD), portions/d	1.10 (0.80)	1.07 (0.79)	1.12 (0.80)	1.10 (0.81)	.12
Vegetable intake, mean (SD), portions/d	2.64 (1.69)	2.67 (1.70)	2.62 (1.67)	2.68 (1.71)	.02
BMI, mean (SD)	24.3 (3.5)	23.6 (3.3)	24.7 (3.4)	26.2 (3.9)	<.001
Total cholesterol, mean (SD), mg/dL	202.1 (36.8)	197.5 (36.3)	204.3 (36.4)	214.3 (42.6)	<.001

Abbreviations: BMI, body mass index (calculated as weight in kilograms divided by height in meters squared); MET, metabolite equivalent of task.

SI conversion factor: To convert total cholesterol to mmol/L, multiply by 0.0259.

^a^ Including retiree, freelance worker, soldier, and homemaker.

### Association of Change in Prediabetes Status With Mortality Rate and Risk of Death

The incidence rate of all-cause mortality per 1000 person-years among individuals who had prediabetes that progressed to diabetes (9.65; 95% CI, 8.18-11.38) was notably higher than those who had persistent prediabetes (4.29; 95% CI, 4.02-4.58) or reverted to normoglycemia (3.26; 95% CI, 2.98-3.56). Similar patterns were observed for the incidence rates of cancer-related mortality (progression to diabetes: 3.22 [95% CI, 2.42-4.28] per 1000 person-years; persistent prediabetes: 1.97 [95% CI, 1.79-2.17] per 1000 person-years; reversion to normoglycemia: 1.41 [95% CI, 1.23-1.62] per 1000 person-years) and CVD-related mortality (progression to diabetes: 2.33 [95% CI, 1.66-3.26] per 1000 person-years; persistent prediabetes: 0.87 [95% CI, 0.75-1.01] per 1000 person-years; reversion to normoglycemia: 0.61 [95% CI, 0.49-0.75] per 1000 person-years) (Table 2; eFigure 2 in Supplement 1).

**Table 2.** Multivariable-Adjusted Hazard Ratios for Associations Between Changes in Prediabetes Status and All-Cause and Cause-Specific Death^a^

**Outcome by change in prediabetes status**	**Cases, No./total No.**	**Mortality rate per 1000 person-years (95% CI)**	**Risk of death, HR (95% CI)**
**All-cause death**			
Reversion to normoglycemia	477/17 021	3.26 (2.98-3.56)	0.99 (0.88-1.10)
Persistent prediabetes	910/26 975	4.29 (4.02-4.58)	1 [Reference]
Progression to diabetes	141/1786	9.65 (8.18-11.38)	1.50 (1.25-1.79)
**Cancer-related death**			
Reversion to normoglycemia	207/17 021	1.41 (1.23-1.62)	0.91 (0.77-1.08)
Persistent prediabetes	417/26 975	1.97 (1.79-2.17)	1 [Reference]
Progression to diabetes	47/1786	3.22 (2.42-4.28)	1.12 (0.83-1.52)
**CVD-related death**			
Reversion to normoglycemia	89/17 021	0.61 (0.49-0.75)	0.97 (0.75-1.25)
Persistent prediabetes	185/26 975	0.87 (0.75-1.01)	1 [Reference]
Progression to diabetes	34/1786	2.33 (1.66-3.26)	1.61 (1.12-2.33)

Abbreviations: CVD, cardiovascular disease; HR, hazard ratio.

^a^ The Cox model were adjusted for age, sex, educational attainment, occupation, marital status, hypertension, total cholesterol level, body mass index, smoking status, alcohol drinking status, vegetable intake, fruit intake, and sugar-sweetened beverage intake.

In the adjusted model, compared with participants with persistent prediabetes, those who experienced progression to diabetes within a 3-year period had a 50% (HR, 1.50; 95% CI, 1.25-1.79) higher risk of all-cause death and a 61% (HR, 1.61; 95% CI, 1.12–2.33) higher risk of CVD-related death. However, reversion to normoglycemia was not associated with the risk of all-cause death (HR, 0.99; 95% CI, 0.88-1.10) or CVD-related death (HR, 0.97; 95% CI, 0.75-1.25). In addition, no association was found between reversion to normoglycemia (HR, 0.91; 95% CI, 0.77-1.08) or progression to diabetes (HR, 1.12; 95% CI, 0.83-1.52) and the risk of cancer-related death.

### Associations of Modifiable Risk Factors With Change in Prediabetes Status

Individuals who were physically active had lower odds of progression to diabetes (OR, 0.88; 95% CI, 0.78-0.99) than individuals who were physically inactive. In addition, those who were physically active had higher odds of reversion to normoglycemia (OR, 1.05; 95% CI, 1.00-1.11) compared with those who were physically inactive (eTable 1 in Supplement 1).

### Joint Associations of Modifiable Risk Factors and Change in Prediabetes Status With Risk of Death

Subsequently, the joint associations of these modifiable risk factors and changes in prediabetes status with the risk of all-cause death were examined (Figure; eTable 2 in Supplement 1). Compared with individuals with persistent prediabetes and physical inactivity, the HRs of all-cause death among individuals who reverted to normoglycemia were 0.72 (95% CI, 0.59-0.87) for those who were active, 0.89 (95% CI, 0.71-1.11) for those who were moderately active, and 1.01 (95% CI, 0.86-1.19) for those who were inactive. The risk of all-cause death varied in those who experienced progression to diabetes depending on whether they were moderately active (HR, 1.00; 95% CI, 0.64-1.56) or active (HR, 1.20; 95% CI, 0.88-1.63) vs inactive (HR, 1.60; 95% CI, 1.25-2.05). Meanwhile, individuals who reverted to normoglycemia and currently smoked had a 60% higher risk of all-cause death (HR, 1.60; 95% CI, 1.31-1.96) compared with individuals with persistent prediabetes who never smoked. Similarly, we found benefits associated with a higher physical activity level (active vs moderately active: HR, 0.71 [95% CI, 0.57-0.88] vs 0.86 [95% CI, 0.68-1.09]) and nonsmoking status (former smoking vs current smoking: HR, 1.60 [95% CI, 1.18-2.16] vs 1.71 [95% CI, 1.32-2.22]) for the risk of all-cause death among those with reversion from prediabetes to normoglycemia (eTable 3 in Supplement 1). Furthermore, among individuals with obesity, the risk of all-cause death varied among those who experienced reversion to normoglycemia and those who had persistent prediabetes. Compared with individuals who had persistent prediabetes and normal weight, the HR of all-cause death among individuals who had obesity and reversion to normoglycemia was 1.10 (95% CI, 0.82-1.49), and the HR among individuals who had obesity and persistent prediabetes was 1.33 (95% CI, 1.10-1.62).

**Figure.**
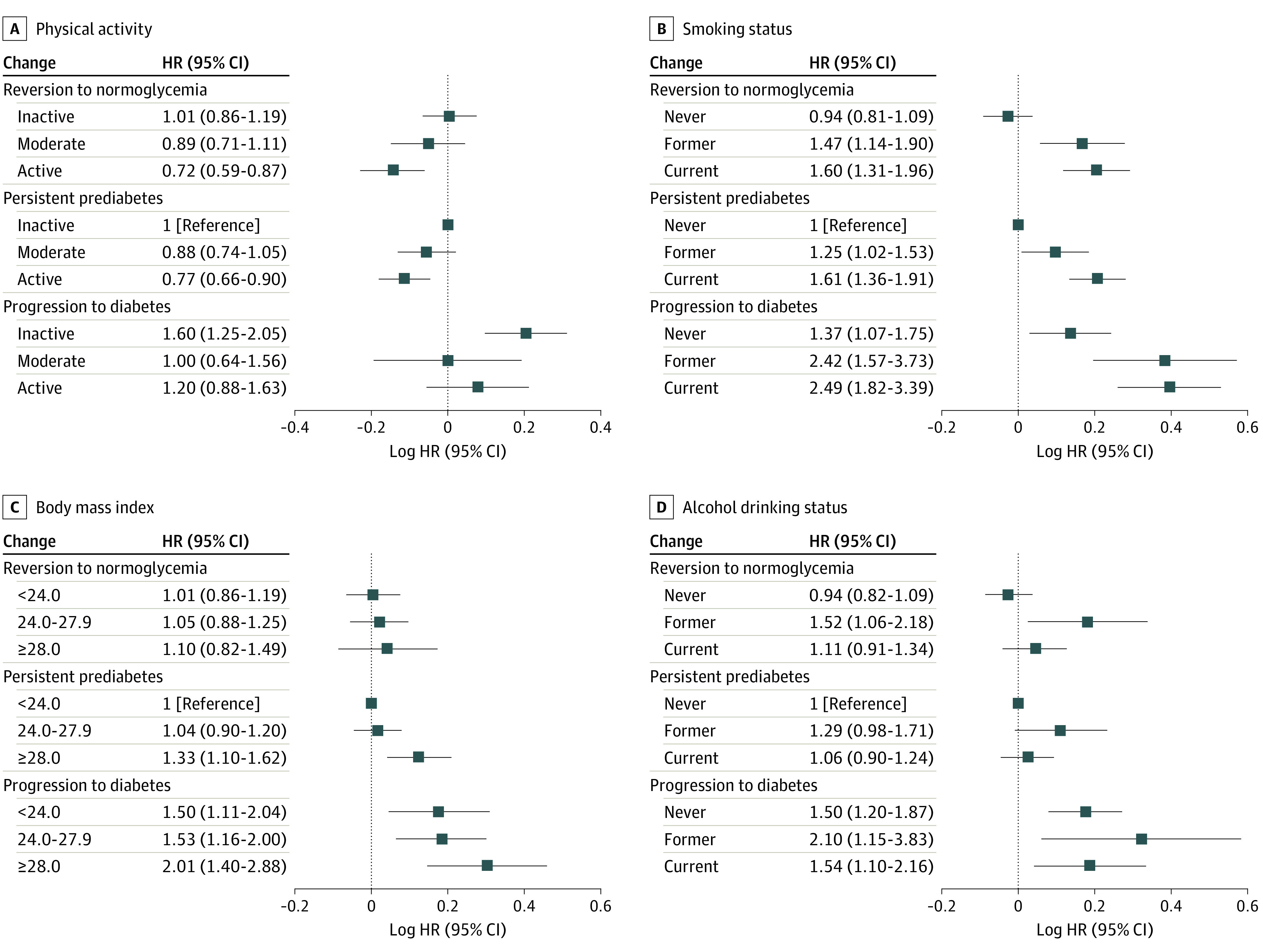
Associations of Changes in Prediabetes Status and Individual Modifiable Risk Factors With Risk of All-Cause Death The Cox models were mutually adjusted for age, sex, educational attainment, occupation, marital status, hypertension, total cholesterol level, body mass index, smoking status, alcohol drinking status, vegetable intake, fruit intake, and sugar-sweetened beverage intake. *P* = .67 for interaction with physical activity, *P* = .54 for interaction with smoking status, *P* = .85 for interaction with body mass index, and *P* = .83 for interaction with alcohol drinking status. HR indicates hazard ratio.

In addition, individuals who reverted to normoglycemia had a life expectancy 2.5 years (95% CI, 1.0-3.9 years) longer if they remained physically active compared with those who had persistent prediabetes and were physically inactive (eFigure 3 in Supplement 1). Compared with individuals with persistent diabetes who never smoked, those with reversion to normoglycemia had a life expectancy 3.6 years (95% CI, 1.8-5.3 years) shorter if they were currently smoking, and those with persistent prediabetes had a life expectancy 3.6 years (95% CI, 2.1-5.1 years) shorter if they were currently smoking.

### Sensitivity Analysis

Sensitivity analyses yielded consistent results (eTables 4-8 in Supplement 1). For all-cause death, the E-value (risk ratio) of the point estimate was 2.37, and the lower confidence bound was 1.81. For CVD-related death, the E-value of the point estimate was 2.60, and the lower confidence bound was 1.49 (eFigure 4 in Supplement 1).

## Discussion

In this large cohort study of 45 782 individuals with prediabetes, we found that reversion from prediabetes to normoglycemia within a 3-year period was not associated with a lower risk of death compared with persistent prediabetes. Interestingly, reversion to normoglycemia combined with the adoption of healthy behaviors, such as a higher level of physical activity and no current smoking, were associated with a substantially lower risk of death and longer life expectancy.

Previous studies^21,22^ have reported increased risks of CVD-related death and all-cause death in those with prediabetes at baseline. The results of our study extended previous findings by confirming that the association between prediabetes and the risk of death might be explained by progression from prediabetes to diabetes. For example, a study of findings from the Hoorn cohort^23^ evaluated the association between changes in prediabetes status and risk of death among 1428 individuals with prediabetes, finding that only participants who progressed from prediabetes to diabetes had a high risk of CVD-related death.

Evidence of the association between reversion from prediabetes to normoglycemia and risk of death has been inconclusive.^9,10,24^ A study of 365 663 participants from South Korea^10^ found that, among individuals with prediabetes that was defined by the FPG level at baseline, early reversion to normoglycemia within 2 to 3 years was associated with a lower risk of CVD-related death. Evidence from the Diabetes Prevention Program Outcomes Study^24^ revealed that reversion to normal glucose regulation, even if transient, was associated with a substantially lower risk of future diabetes. However, there have also been counterintuitive findings suggesting that reversion from prediabetes to normoglycemia may not be associated with a lower risk of death. For instance, a Korean cohort study^9^ of 61 927 participants with prediabetes that was defined by FPG level found that reversion to normoglycemia within 2 years was not associated with cardiovascular outcomes and all-cause mortality. In the present study, we had a sufficient sample size and a more specific target population (ie, only participants with prediabetes status at enrollment were included), and we still found that reversion from prediabetes to normoglycemia was not associated with a lower risk of death compared with persistent prediabetes.

Notably, we aimed to identify factors that might explain why participants with reversion from prediabetes to normoglycemia did not have lower risk of death compared with those with persistent prediabetes. We hypothesized that different behavioral patterns may be the reason. Our study found that individuals with prediabetes status who remained physically active or never smoked had substantial reductions in the risk of death. Moreover, our study revealed that BMI was an important risk factor associated with changes in prediabetes status. Previous studies^25,26^ suggested that the transition to metabolic unhealthiness (such as the onset of type 2 diabetes) substantially increases the risk of death among individuals with obesity. Because several other metabolic health factors, such as hypertension and hyperlipidemia, might confound the association between changes in prediabetes status and risk of death, we further adjusted for hypertension and total cholesterol level and found no substantial differences in the results.

Previous evidence^27^ suggested that progression from prediabetes to diabetes was associated with severe deterioration of β-cell dysfunction, while successful lifestyle modification was associated with improvements in insulin sensitivity and β-cell function. For instance, a growing body of evidence analyzed in a review^28^ has revealed the benefits of lifestyle modification, such as regular physical activity, healthy diet, and normal weight, for prediabetes stabilization and reversion from prediabetes to normoglycemia. Likewise, our study found that being physically active was beneficial for reversion from prediabetes to normoglycemia. Moreover, the potential reason underlying the benefits of reversion from prediabetes to normoglycemia for the risk of death was primarily lifestyle modification.^29^

### Strengths and Limitations

This study has several strengths. The study is well characterized, with a large cohort of individuals with prediabetes. Furthermore, the mortality data were obtained from death registry records; therefore, few individuals were unavailable for follow-up.

The study also has several limitations. First, the definition of prediabetes was based on a 1-time FPG assessment due to the lack of availability of an oral glucose tolerance test. Of note, the implications of impaired fasting glucose and impaired glucose tolerance for cardiovascular outcomes were similar.^30^ Because an individual’s FPG level changes over time, a potential measurement error in FPG assessment may lead to overestimation of the number of participants who reverted to normoglycemia.^31^ Second, similar to any observational study, although we adjusted for potential confounding factors, we could not completely exclude reverse causality or unmeasured confounders. Third, we assessed changes in prediabetes status within a 3-year period because it is common during a clinical visit for patients to be interested in knowing their risk of death according to the change in their prediabetes status compared with previous visits. Although treating change as a time-varying factor may further reveal the association of change in prediabetes status with mortality, the approach requires large samples due to the exponentially increasing number of combinations of exposure levels over follow-up. Fourth, the Taiwan MJ cohort consists of Asian individuals who attended clinical wellness visits and generally had higher socioeconomic status. Thus, our findings may be generalizable only to Asian populations with similar socioeconomic backgrounds.

## Conclusions

In this cohort study, reversion to normoglycemia within a 3-year period was not associated with the overall risk of death among participants with prediabetes. Reversion to normoglycemia in combination with physically activity was associated with a lower risk of death compared with persistent prediabetes and physical inactivity, and risk of death varied between those who experienced reversion to normoglycemia and those who had persistent prediabetes among individuals with obesity. These findings highlight the importance of lifestyle modifications among individuals with prediabetes status. The development of comprehensive personalized intervention strategies for lifestyle modification should be encouraged among this target population.

## Notes

Supplement 1. **eTable 1.** Role of Modifiable Risk Factors in Change in Prediabetes Status **eTable 2.** Joint Association of Change in Prediabetes Status and Individual Modifiable Risk Factors With Risk of All-Cause Death **eTable 3.** Role of Modifiable Risk Factors in All-Cause Death Among Participants With Reversion From Prediabetes Status to Normoglycemia **eTable 4.** Risk of All-Cause Death According to Change in Prediabetes Status Defined by WHO Criteria **eTable 5.** Hazard Ratios for All-Cause and Cause-Specific Death by Change in Prediabetes Status After Additional Adjustment for Antihypertensive and Lipid-Lowering Medications **eTable 6.** Hazard Ratios for All-Cause and Cause-Specific Death by Change in Prediabetes Status Using Competing Risk Model **eTable 7.** Hazard Ratios of All-Cause and Cause-Specific Death by Change in Prediabetes Status After Excluding First 2 Years of Deaths During Follow-up Period **eTable 8.** Hazard Ratios of All-Cause and Cause-Specific Death by Change in Prediabetes Status in Complete-Case Analysis **eFigure 1.** Flowchart of Study Design **eFigure 2.** Incidence Rate per 1000 Person-years of All-Cause and Cause-Specific Death by Change in Prediabetes Status **eFigure 3.** Difference in Life Expectancy For Modifiable Risk Factors Among Participants With Persistent Prediabetes or Reversion to Normoglycemia **eFigure 4.** E-Value for Associations of Progression From Prediabetes to Diabetes With All-Cause and CVD-Related Death

Supplement 2.
Data Sharing Statement

## References

[1] Tabák AG, Herder C, Rathmann W, Brunner EJ, Kivimäki M. Prediabetes: a high-risk state for diabetes development. Lancet. 2012;379(9833):2279-2290. doi:10.1016/S0140-6736(12)60283-9 22683128 PMC3891203

[2] International Diabetes Federation. IDF Diabetes Atlas. 8th ed. International Diabetes Federation; 2017.

[3] Wang L, Peng W, Zhao Z, . Prevalence and treatment of diabetes in China, 2013-2018. JAMA. 2021;326(24):2498-2506. doi:10.1001/jama.2021.22208 34962526 PMC8715349

[4] Li Y, Teng D, Shi X, . Prevalence of diabetes recorded in mainland China using 2018 diagnostic criteria from the American Diabetes Association: national cross sectional study. BMJ. 2020;369:m997. doi:10.1136/bmj.m997 32345662 PMC7186854

[5] Schlesinger S, Neuenschwander M, Barbaresko J, . Prediabetes and risk of mortality, diabetes-related complications and comorbidities: umbrella review of meta-analyses of prospective studies. Diabetologia. 2022;65(2):275-285. doi:10.1007/s00125-021-05592-3 34718834 PMC8741660

[6] Hostalek U. Global epidemiology of prediabetes—present and future perspectives. Clin Diabetes Endocrinol. 2019;5:5. doi:10.1186/s40842-019-0080-0 31086677 PMC6507173

[7] Veronese N, Noale M, Sinclair A, . Risk of progression to diabetes and mortality in older people with prediabetes: the English Longitudinal Study on Ageing. Age Ageing. 2022;51(2):afab222. doi:10.1093/ageing/afab222 35134845 PMC8824760

[8] Liu X, Wu S, Song Q, Wang X. Reversion from pre–diabetes mellitus to normoglycemia and risk of cardiovascular disease and all-cause mortality in a Chinese population: a prospective cohort study. J Am Heart Assoc. 2021;10(3):e019045. doi:10.1161/JAHA.120.019045 33496188 PMC7955447

[9] Lee G, Kim SM, Choi S, . The effect of change in fasting glucose on the risk of myocardial infarction, stroke, and all-cause mortality: a nationwide cohort study. Cardiovasc Diabetol. 2018;17(1):51. doi:10.1186/s12933-018-0694-z 29626936 PMC5889526

[10] Kim SM, Lee G, Choi S, . Association of early-onset diabetes, prediabetes and early glycaemic recovery with the risk of all-cause and cardiovascular mortality. Diabetologia. 2020;63(11):2305-2314. doi:10.1007/s00125-020-05252-y 32820349

[11] Vistisen D, Kivimäki M, Perreault L, . Reversion from prediabetes to normoglycaemia and risk of cardiovascular disease and mortality: the Whitehall II cohort study. Diabetologia. 2019;62(8):1385-1390. doi:10.1007/s00125-019-4895-0 31123789 PMC6647230

[12] Wu X, Tsai SP, Tsao CK, . Cohort profile: the Taiwan MJ cohort: half a million Chinese with repeated health surveillance data. Int J Epidemiol. 2017;46(6):1744. doi:10.1093/ije/dyw282 28204597

[13] Wen CP, Wai JPM, Tsai MK, . Minimum amount of physical activity for reduced mortality and extended life expectancy: a prospective cohort study. Lancet. 2011;378(9798):1244-1253. doi:10.1016/S0140-6736(11)60749-6 21846575

[14] Wen CP, Cheng TYD, Tsai MK, . All-cause mortality attributable to chronic kidney disease: a prospective cohort study based on 462 293 adults in Taiwan. Lancet. 2008;371(9631):2173-2182. doi:10.1016/S0140-6736(08)60952-6 18586172

[15] Tu H, Wen CP, Tsai SP, . Cancer risk associated with chronic diseases and disease markers: prospective cohort study. BMJ. 2018;360:k134. doi:10.1136/bmj.k134 29386192 PMC5791146

[16] American Diabetes Association. 2. Classification and diagnosis of diabetes: *Standards of Medical Care in Diabetes–2021*. Diabetes Care. 2021;44(suppl 1):S15-S33. doi:10.2337/dc21-S002 33298413

[17] Martinez-Gomez D, Esteban-Cornejo I, Lopez-Garcia E, . Physical activity less than the recommended amount may prevent the onset of major biological risk factors for cardiovascular disease: a cohort study of 198 919 adults. Br J Sports Med. 2020;54(4):238-244. doi:10.1136/bjsports-2018-099740 30554146

[18] Dehbi HM, Royston P, Hackshaw A. Life expectancy difference and life expectancy ratio: two measures of treatment effects in randomised trials with non-proportional hazards. BMJ. 2017;357:j2250. doi:10.1136/bmj.j2250 28546261 PMC5444092

[19] World Health Organization & International Diabetes Federation. Definition and Diagnosis of Diabetes Mellitus and Intermediate Hyperglycaemia: Report of a WHO/IDF Consultation. World Health Organization; 2006. Accessed October 15, 2021. https://apps.who.int/iris/handle/10665/43588

[20] VanderWeele TJ, Ding P. Sensitivity analysis in observational research: introducing the E-value. Ann Intern Med. 2017;167(4):268-274. doi:10.7326/M16-2607 28693043

[21] Islam Z, Akter S, Inoue Y, ; Japan Epidemiology Collaboration on Occupational Health Study Group. Prediabetes, diabetes, and the risk of all-cause and cause-specific mortality in a Japanese working population: Japan Epidemiology Collaboration on Occupational Health study. Diabetes Care. 2021;44(3):757-764. doi:10.2337/dc20-1213 33441421 PMC7896260

[22] Vistisen D, Witte DR, Brunner EJ, . Risk of cardiovascular disease and death in individuals with prediabetes defined by different criteria: the Whitehall II study. Diabetes Care. 2018;41(4):899-906. doi:10.2337/dc17-2530 29453200 PMC6463620

[23] Rijkelijkhuizen JM, Nijpels G, Heine RJ, Bouter LM, Stehouwer CDA, Dekker JM. High risk of cardiovascular mortality in individuals with impaired fasting glucose is explained by conversion to diabetes: the Hoorn study. Diabetes Care. 2007;30(2):332-336. doi:10.2337/dc06-1238 17259503

[24] Perreault L, Pan Q, Mather KJ, Watson KE, Hamman RF, Kahn SE; Diabetes Prevention Program Research Group. Effect of regression from prediabetes to normal glucose regulation on long-term reduction in diabetes risk: results from the Diabetes Prevention Program Outcomes Study. Lancet. 2012;379(9833):2243-2251. doi:10.1016/S0140-6736(12)60525-X 22683134 PMC3555407

[25] Zembic A, Eckel N, Stefan N, Baudry J, Schulze MB. An empirically derived definition of metabolically healthy obesity based on risk of cardiovascular and total mortality. JAMA Netw Open. 2021;4(5):e218505. doi:10.1001/jamanetworkopen.2021.8505 33961036 PMC8105750

[26] Lee J, Kwak SY, Park D, Kim GE, Park CY, Shin MJ. Prolonged or transition to metabolically unhealthy status, regardless of obesity status, is associated with higher risk of cardiovascular disease incidence and mortality in Koreans. Nutrients. 2022;14(8):1644. doi:10.3390/nu14081644 35458208 PMC9028697

[27] Snehalatha C, Mary S, Selvam S, . Changes in insulin secretion and insulin sensitivity in relation to the glycemic outcomes in subjects with impaired glucose tolerance in the Indian Diabetes Prevention Programme–1 (IDPP-1). Diabetes Care. 2009;32(10):1796-1801. doi:10.2337/dc09-0676 19587369 PMC2752907

[28] Sallar A, Dagogo-Jack S. Regression from prediabetes to normal glucose regulation: state of the science. Exp Biol Med (Maywood). 2020;245(10):889-896. doi:10.1177/1535370220915644 32212859 PMC7268926

[29] Lawal Y, Bello F, Kaoje YS. Prediabetes deserves more attention: a review. Clin Diabetes. 2020;38(4):328-338. doi:10.2337/cd19-0101 33132502 PMC7566925

[30] Huang Y, Cai X, Mai W, Li M, Hu Y. Association between prediabetes and risk of cardiovascular disease and all cause mortality: systematic review and meta-analysis. BMJ. 2016;355:i5953. doi:10.1136/bmj.i5953 27881363 PMC5121106

[31] Daves M, Cemin R, Fattor B, . Evaluation of hematocrit bias on blood glucose measurement with six different portable glucose meters. Biochem Med (Zagreb). 2011;21(3):306-311. doi:10.11613/BM.2011.041 22420245

