# Supplementary material for: Risk of Death Associated With Reversion From Prediabetes to Normoglycemia and the Role of Modifiable Risk Factors
Source: JAMA Netw Open. 2023 Mar 28;6(3):e234989. doi: 10.1001/jamanetworkopen.2023.4989 (PMC10051049; doi:10.1001/jamanetworkopen.2023.4989)
Supplement: Supplement 1. — eTable 1. Role of Modifiable Risk Factors in Change in Prediabetes Status eTable 2. Joint Association of Change in Prediabetes Status and Individual Modifiable Risk Factors With Risk of All-Cause Death eTable 3. Role of Modifiable Risk Factors in All-Cause Death Among Participants With Reversion From Prediabetes Status to Normoglycemia eTable 4. Risk of All-Cause Death According to Change in Prediabetes Status Defined by WHO Criteria eTable 5. Hazard Ratios for All-Cause and Cause-Specific Death by Change in Prediabetes Status After Additional Adjustment for Antihypertensive and Lipid-Lowering Medications eTable 6. Hazard Ratios for All-Cause and Cause-Specific Death by Change in Prediabetes Status Using Competing Risk Model eTable 7. Hazard Ratios of All-Cause and Cause-Specific Death by Change in Prediabetes Status After Excluding First 2 Years of Deaths During Follow-up Period eTable 8. Hazard Ratios of All-Cause and Cause-Specific Death by Change in Prediabetes Status in Complete-Case Analysis eFigure 1. Flowchart of Study Design eFigure 2. Incidence Rate per 1000 Person-years of All-Cause and Cause-Specific Death by Change in Prediabetes Status eFigure 3. Difference in Life Expectancy For Modifiable Risk Factors Among Participants With Persistent Prediabetes or Reversion to Normoglycemia eFigure 4. E-Value for Associations of Progression From Prediabetes to Diabetes With All-Cause and CVD-Related Death [file jamanetwopen-e234989-s001.pdf]

Supplementary Online Content

Cao Z, Li W, Wen CP, et al. Risk of death associated with reversion from prediabetes to normoglycemia and the role of modifiable risk factors. *JAMA Netw Open*. 2023;6(3):e234989. doi:10.1001/jamanetworkopen.2023.4989

eTable 1. Role of Modifiable Risk Factors in Change in Prediabetes Status

eTable 2. Joint Association of Change in Prediabetes Status and Individual Modifiable Risk Factors With Risk of All-Cause Death

eTable 3. Role of Modifiable Risk Factors in All-Cause Death Among Participants With Reversion From Prediabetes Status to Normoglycemia

eTable 4. Risk of All-Cause Death According to Change in Prediabetes Status Defined by WHO Criteria

eTable 5. Hazard Ratios for All-Cause and Cause-Specific Death by Change in Prediabetes Status After Additional Adjustment for Antihypertensive and Lipid-Lowering Medications

eTable 6. Hazard Ratios for All-Cause and Cause-Specific Death by Change in Prediabetes Status Using Competing Risk Model

eTable 7. Hazard Ratios of All-Cause and Cause-Specific Death by Change in Prediabetes Status After Excluding First 2 Years of Deaths During Follow-up Period

eTable 8. Hazard Ratios of All-Cause and Cause-Specific Death by Change in Prediabetes Status in Complete-Case Analysis

eFigure 1. Flowchart of Study Design

eFigure 2. Incidence Rate per 1000 Person-years of All-Cause and Cause-Specific Death by Change in Prediabetes Status

eFigure 3. Difference in Life Expectancy For Modifiable Risk Factors Among Participants With Persistent Prediabetes or Reversion to Normoglycemia

eFigure 4. E-Value for Associations of Progression From Prediabetes to Diabetes With All-Cause and CVD-Related Death

This supplemental material has been provided by the authors to give readers additional information about their work.

© 2023 Cao Z et al. *JAMA Network Open*.

eTable 1. Role of Modifiable Risk Factors in Change in Prediabetes Status

Modifiable risk factors	Persistent prediabetes	Reversion to normoglycemia		Progression to diabetes	
	n (%)	n (%)	OR (95% CI)	n (%)	OR (95% CI)
**Physical activity**					
Inactive	12,727 (47.2)	8,372 (49.2)	1 (Reference)	895 (50.1)	1 (Reference)
Moderate	6,012 (22.3)	3,998 (23.5)	1.05 (1.00-1.10)	354 (19.8)	0.90 (0.79-1.02)
Active	8,236 (30.5)	4,651 (27.3)	1.05 (1.00-1.11)	537 (30.1)	0.88 (0.78-0.99)
**Smoking status**					
Never	17,993 (66.7)	11,761 (69.1)	1 (Reference)	1,144 (64.1)	1 (Reference)
Former	2,446 (9.1)	1,254 (7.4)	1.05 (0.98-1.14)	160 (9.0)	1.05 (0.87-1.27)
Current	6,536 (24.2)	4,006 (23.5)	1.11 (1.05-1.18)	482 (27.0)	1.41 (1.23-1.61)
**Drinking status**					
Never	19,873 (73.7)	13,133 (77.2)	1 (Reference)	1,280 (71.7)	1 (Reference)
Former	793 (2.9)	429 (2.5)	1.04 (0.91-1.17)	67 (3.7)	1.07 (0.82-1.40)
Current	6,309 (23.4)	3,459 (20.3)	0.95 (0.90-1.00)	439 (24.6)	0.99 (0.87-1.13)
**Body mass index**					
Normal	12,034 (44.6)	9,769 (57.4)	1 (Reference)	495 (27.7)	1 (Reference)
Overweight	11,072 (41.1)	5,677 (33.3)	0.74 (0.71-0.77)	805 (45.1)	1.61 (1.43-1.81)
Obesity	3,869 (14.3)	1,575 (9.3)	0.56 (0.53-0.60)	486 (27.2)	2.70 (2.35-3.09)
**Fruit intake**					
Seldom	8,637 (32.0)	5,875 (34.5)	1 (Reference)	587(32.9)	1 (Reference)
Moderate	15,795 (58.5)	9,761 (57.3)	0.95 (0.91-0.99)	1,036 (58.0)	0.97 (0.87-1.09)
Frequent	2,543 (9.5)	1,385 (8.1)	0.82 (0.76-0.89)	163 (9.1)	1.06 (0.88-1.29)
**Vegetable intake**					
Seldom	2,130 (7.9)	1,330 (7.8)	1 (Reference)	145 (8.1)	1 (Reference)
Moderate	14,525 (53.8)	8,874 (52.1)	1.00 (0.93-1.08)	905 (50.7)	0.95 (0.79-1.15)
Frequent	10,320 (38.3)	6,817 (40.1)	1.11 (1.03-1.20)	736 (41.2)	0.99 (0.82-1.20)
**Sugar-sweetened beverages**					
Never	11,118 (41.2)	6,965 (40.9)	1 (Reference)	879 (49.2)	1 (Reference)
Seldom	7,519 (27.9)	4,633 (27.2)	0.87 (0.83-0.92)	449 (25.1)	0.95 (0.84-1.07)
Frequent	8,338 (30.9)	5,423 (31.9)	0.88 (0.83-0.92)	458 (25.6)	0.94 (0.83-1.07)

Multinomial logistic regression models were used to obtain the OR and 95% CIs, and adjusted for age, sex, educational attainment, occupation status, marriage status, hypertension, total cholesterol, as well as smoking status, alcohol intake frequency, body mass index, vegetable intake, fruit intake, sugar-sweetened beverages were adjusted mutually.

© 2023 Cao Z et al. *JAMA Network Open*.

eTable 2. Joint Association of Change in Prediabetes Status and Individual Modifiable Risk Factors With Risk of All-Cause Death

Modifiable risk factors	Change of prediabetes			*P* for interaction
	Reversion to normoglycemia	Persistent prediabetes	Progression to diabetes	
**Physical activity**				0.67
Inactive	1.01 (0.86-1.19)	1 (Reference)	1.60 (1.25-2.05)	
Moderate	0.89 (0.71-1.11)	0.88 (0.74-1.05)	1.00 (0.64-1.56)	
Active	0.72 (0.59-0.87)	0.77 (0.66-0.90)	1.20 (0.88-1.63)	
**Smoking status**				0.54
Never	0.94 (0.81-1.09)	1 (Reference)	1.37 (1.07-1.75)	
Former	1.47 (1.14-1.90)	1.25 (1.02-1.53)	2.42 (1.57-3.73)	
Current	1.60 (1.31-1.96)	1.61 (1.36-1.91)	2.49 (1.82-3.39)	
**Body mass index**				0.85
Normal	1.01 (0.86-1.19)	1 (Reference)	1.50 (1.11-2.04)	
Overweight	1.05 (0.88-1.25)	1.04 (0.90-1.20)	1.53 (1.16-2.00)	
Obesity	1.10 (0.82-1.49)	1.33 (1.10-1.62)	2.01 (1.40-2.88)	
**Drinking status**				0.83
Never	0.94 (0.82-1.09)	1 (Reference)	1.50 (1.20-1.87)	
Former	1.52 (1.06-2.18)	1.29 (0.98-1.71)	2.10 (1.15-3.83)	
Current	1.11 (0.91-1.34)	1.06 (0.90-1.24)	1.54 (1.10-2.16)	
**Fruit intake**				0.50
Frequent (> 2 serving/day)	0.60 (0.32-1.11)	1 (Reference)	1.50 (0.68-3.32)	
Moderate (1-2 serving/day)	0.92 (0.67-1.26)	0.88 (0.65-1.20)	1.34 (0.93-1.92)	
Seldom (< 1 serving/day)	0.99 (0.71-1.37)	1.04 (0.76-1.42)	1.52 (1.01-2.29)	
**Vegetable intake**				0.11
Frequent (> 2 serving/day)	0.97 (0.83-1.15)	1 (Reference)	1.92 (1.50-2.45)	
Moderate (1-2 serving/day)	1.06 (0.90-1.26)	1.06 (0.92-1.21)	1.27 (0.95-1.69)	
Seldom (< 1 serving/day)	1.25 (0.91-1.74)	1.30 (1.01-1.67)	1.45 (0.79-2.65)	
**Sugar-sweetened beverages**				0.57
Never	0.93 (0.81-1.07)	1 (Reference)	1.51 (1.21-1.89)	
Seldom (1-3 cups/week)	0.92 (0.73-1.16)	0.88 (0.74-1.05)	1.20 (0.78-1.84)	
Frequent (> 3 serving/week)	0.98 (0.77-1.23)	0.83 (0.69-1.00)	1.31 (0.91-1.90)	

The Cox models were adjusted for age, sex, educational attainment, occupation status, marriage status, hypertension, total cholesterol, as well as smoking status, alcohol intake frequency, body mass index, vegetable intake, fruit intake, sugar-sweetened beverages were adjusted mutually.

© 2023 Cao Z et al. *JAMA Network Open*.

eTable 3. Role of Modifiable Risk Factors in All-Cause Death Among Participants With Reversion From Prediabetes Status to Normoglycemia

Modifiable risk factors	HR (95% CI)	P value
**Physical activity**		
Inactive	1 (Reference)	
Moderate	0.86 (0.68-1.09)	0.222
Active	0.71 (0.57-0.88)	0.002
**Smoking status**		
Never	1 (Reference)	
Former	1.60 (1.18-2.16)	0.002
Current	1.71 (1.32-2.22)	<0.001
**Drinking status**		
Never	1 (Reference)	
Former	1.66 (1.13-2.45)	0.010
Current	1.22 (0.97-1.54)	0.085
**Body mass index**		
Normal	1 (Reference)	
Overweight	1.04 (0.85-1.26)	0.696
Obesity	1.08 (0.79-1.48)	0.622
**Fruit intake**		
Seldom	1 (Reference)	
Moderate	0.95 (0.78-1.15)	0.617
Frequent	0.61 (0.34-1.08)	0.088
**Vegetable intake**		
Seldom	1 (Reference)	
Moderate	0.86 (0.61-1.20)	0.373
Frequent	0.78 (0.55-1.11)	0.169
**Sugar-sweetened beverages**		
Never	1 (Reference)	
Seldom	1.00 (0.78-1.28)	0.979
Frequent	1.06 (0.82-1.36)	0.672

The Cox models were adjusted for age, sex, educational attainment, occupation status, marriage status, hypertension, total cholesterol, as well as smoking status, alcohol intake frequency, body mass index, vegetable intake, fruit intake, sugar-sweetened beverages were adjusted mutually.

© 2023 Cao Z et al. *JAMA Network Open*.

eTable 4. Risk of All-Cause Death According to Change in Prediabetes Status Defined by WHO Criteria (n = 9531)

All-cause mortality	Persistent prediabetes	Reversion to normoglycemia	Progression to diabetes
Cases/total	164/3580	222/4728	100/1123
HR (95% CI)			
Model 1	1 (Reference)	1.13 (0.93-1.39)	1.55 (1.21-1.99)
Model 2	1 (Reference)	1.14 (0.93-1.39)	1.54 (1.20-1.97)
Model 3	1 (Reference)	1.17 (0.96-1.44)	1.53 (1.19-1.97)

Model 1 was adjusted for age and sex; Model 2 was adjusted for age, sex, educational attainment, occupation status, marriage status; Model 3 was adjusted for age, sex, educational attainment, occupation status, marriage status, smoking status, alcohol intake frequency, body mass index, physical activity, sugar-sweetened beverages, hypertension, total cholesterol.

© 2023 Cao Z et al. *JAMA Network Open*.

eTable 5. Hazard Ratios for All-Cause and Cause-Specific Death by Change in Prediabetes Status After Additional Adjustment for Antihypertensive and Lipid-Lowering Medications

Outcomes	Change of prediabetes		
	Progression to diabetes	Persistent prediabetes	Reversion to normoglycemia
All-cause mortality	1.47 (1.23-1.76)	1 (Reference)	0.98 (0.88-1.10)
Cause-specific mortality			
Cancer mortality	1.12 (0.83-1.52)	1 (Reference)	0.91 (0.77-1.08)
CVD mortality	1.53 (1.06-2.21)	1 (Reference)	0.96 (0.75-1.25)

The analysis was adjusted for age, sex, educational attainment, occupation status, marriage status, smoking status, alcohol intake frequency, body mass index, physical activity, sugar-sweetened beverages, hypertension, total cholesterol, anti-hypertensive and lipid-lowering medication.

© 2023 Cao Z et al. *JAMA Network Open*.

eTable 6. Hazard Ratios for All-Cause and Cause-Specific Death by Change in Prediabetes Status Using Competing Risk Model

**Change of prediabetes**	Hazard Ratio (95% CI)			
	Model 1	Model 2	Model 3	Model 4
**Cancer-related mortality**				
Persistent prediabetes	1 (Reference)	1 (Reference)	1 (Reference)	1 (Reference)
Reversion to normoglycemia	0.93 (0.78-1.09)	0.92 (0.78-1.09)	0.91 (0.77-1.08)	0.91 (0.77-1.080
Progression to diabetes	1.14 (0.84-1.55)	1.12 (0.82-1.51)	1.09 (0.80-1.48)	1.09 (0.80-1.48)
**CVD-related mortality**				
Persistent prediabetes	1 (Reference)	1 (Reference)	1 (Reference)	1 (Reference)
Reversion to normoglycemia	0.96 (0.74-1.23)	0.95 (0.74-1.23)	0.98 (0.76-1.26)	0.98 (0.76-1.26)
Progression to diabetes	1.78 (1.23-2.58)	1.74 (1.20-2.52)	1.56 (1.07-2.27)	1.49 (1.03-2.16)

Model 1 was adjusted for age and sex; Model 2 was adjusted for age, sex, educational attainment, occupation status, marriage status; Model 3 was adjusted for age, sex, educational attainment, occupation status, marriage status, smoking status, alcohol intake frequency, body mass index, physical activity, sugar-sweetened beverages, hypertension, total cholesterol. Model 4 was adjusted for age, sex, educational attainment, occupation status, marriage status, smoking status, alcohol intake frequency, body mass index, physical activity, sugar-sweetened beverages, hypertension, total cholesterol, anti-hypertensive and lipid-lowering medication.

© 2023 Cao Z et al. *JAMA Network Open*.

eTable 7. Hazard Ratios of All-Cause and Cause-Specific Death by Change in Prediabetes Status After Excluding First 2 Years of Deaths During Follow-up Period

Change of prediabetes	Deaths	Hazard Ratio (95% CI)			
		Model 1	Model 2	Model 3	Model 4
**All-cause mortality**					
Persistent prediabetes	910	1 (Reference)	1 (Reference)	1 (Reference)	1 (Reference)
Reversion to normoglycemia	477	1.00 (0.89-1.12)	0.99 (0.88-1.11)	0.99 (0.88-1.11)	0.99 (0.88-1.11)
Progression to diabetes	141	1.54 (1.27-1.86)	1.51 (1.25-1.83)	1.44 (1.19-1.74)	1.40 (1.15-1.70)
**Cancer-related mortality**					
Persistent prediabetes	417	1 (Reference)	1 (Reference)	1 (Reference)	1 (Reference)
Reversion to normoglycemia	207	0.95 (0.80-1.13)	0.95 (0.82-1.57)	0.94 (0.79-1.12)	0.94 (0.79-1.12)
Progression to diabetes	47	1.16 (0.84-1.60)	1.13 (0.80-1.13)	1.10 (0.79-1.52)	1.10 (0.79-1.52)
**CVD-related mortality**					
Persistent prediabetes	185	1 (Reference)	1 (Reference)	1 (Reference)	1 (Reference)
Reversion to normoglycemia	89	0.97 (0.75-1.27)	0.96 (0.74-1.26)	0.99 (0.75-1.29)	0.98 (0.75-1.28)
Progression to diabetes	34	1.76 (1.18-2.61)	1.72 (1.16-2.55)	1.56 (1.05-2.32)	1.47 (0.99-2.20)

Model 1 was adjusted for age and sex; Model 2 was adjusted for age, sex, educational attainment, occupation status, marriage status; Model 3 was adjusted for age, sex, educational attainment, occupation status, marriage status, smoking status, alcohol intake frequency, body mass index, physical activity, sugar-sweetened beverages, hypertension, total cholesterol. Model 4 was adjusted for age, sex, educational attainment, occupation status, marriage status, smoking status, alcohol intake frequency, body mass index, physical activity, sugar-sweetened beverages, hypertension, total cholesterol, anti-hypertensive and lipid-lowering medication.

© 2023 Cao Z et al. *JAMA Network Open*.

eTable 8. Hazard Ratios of All-Cause and Cause-Specific Death by Change in Prediabetes Status in Complete-Case Analysis

**Change of prediabetes**	Deaths	Hazard Ratio (95% CI)			
		Model 1	Model 2	Model 3	Model 4
**All-cause mortality**					
Persistent prediabetes	670	1 (Reference)	1 (Reference)	1 (Reference)	1 (Reference)
Reversion to normoglycemia	337	0.98 (0.86-1.12)	0.97 (0.85-1.11)	0.96 (0.84-1.78)	0.96 (0.84-1.10)
Progression to diabetes	100	1.55 (1.26-1.91)	1.52 (1.23-1.87)	1.44 (1.16-1.78)	1.41 (1.14-1.74)
**Cancer-related mortality**					
Persistent prediabetes	310	1 (Reference)	1 (Reference)	1 (Reference)	1 (Reference)
Reversion to normoglycemia	154	0.95 (0.78-1.16)	1.09 (0.76-1.56)	0.92 (0.76-1.12)	0.92 (0.76-1.12)
Progression to diabetes	33	1.11 (0.78-1.60)	0.95 (0.78-1.16)	1.06 (0.74-1.52)	1.06 (0.74-1.52)
**CVD-related mortality**					
Persistent prediabetes	132	1 (Reference)	1 (Reference)	1 (Reference)	1 (Reference)
Reversion to normoglycemia	52	0.80 (0.58-1.11)	0.80 (0.58-1.11)	0.82 (0.59-1.14)	0.81 (0.59-1.12)
Progression to diabetes	19	1.43 (0.89-2.32)	1.41 (0.87-2.28)	1.22 (0.75-1.98)	1.16 (0.71-1.89)

Model 1 was adjusted for age and sex; Model 2 was adjusted for age, sex, educational attainment, occupation status, marriage status; Model 3 was adjusted for age, sex, educational attainment, occupation status, marriage status, smoking status, alcohol intake frequency, body mass index, physical activity, sugar-sweetened beverages, hypertension, total cholesterol. Model 4 was adjusted for age, sex, educational attainment, occupation status, marriage status, smoking status, alcohol intake frequency, body mass index, physical activity, sugar-sweetened beverages, hypertension, total cholesterol, anti-hypertensive and lipid-lowering medication.

© 2023 Cao Z et al. *JAMA Network Open*.

eFigure 1. Flowchart of Study Design

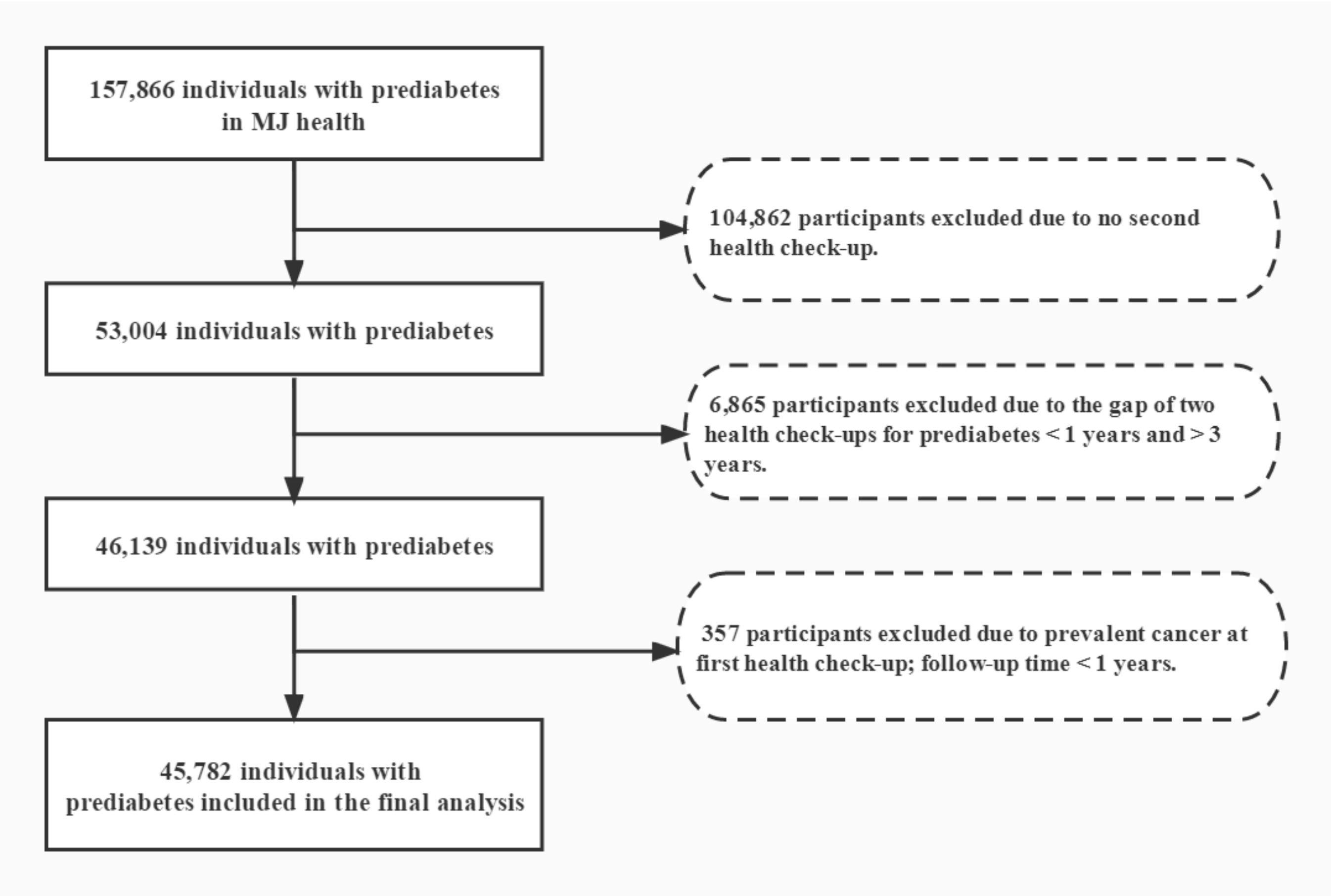

© 2023 Cao Z et al. *JAMA Network Open*.

eFigure 2. Incidence Rate per 1000 Person-years of All-Cause and Cause-Specific Death by Change in Prediabetes Status

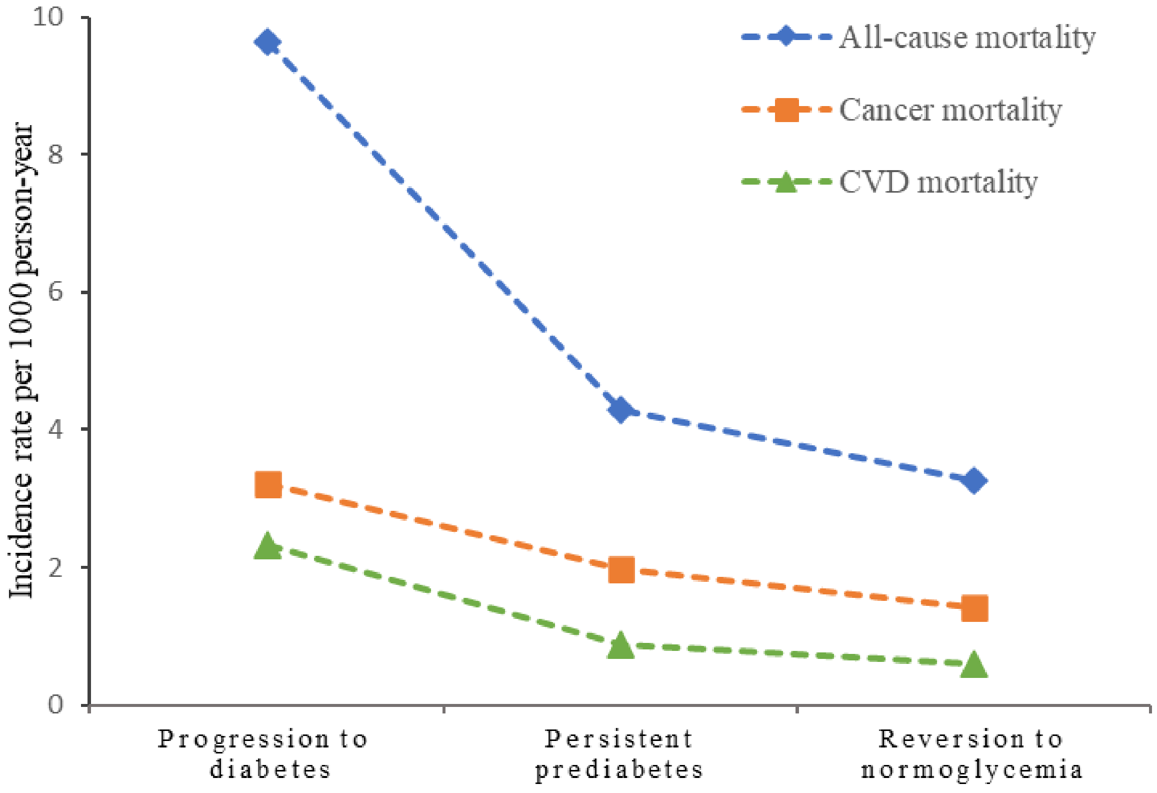

© 2023 Cao Z et al. *JAMA Network Open*.

eFigure 3. Difference in Life Expectancy For Modifiable Risk Factors Among Participants With Persistent Prediabetes or Reversion to Normoglycemia

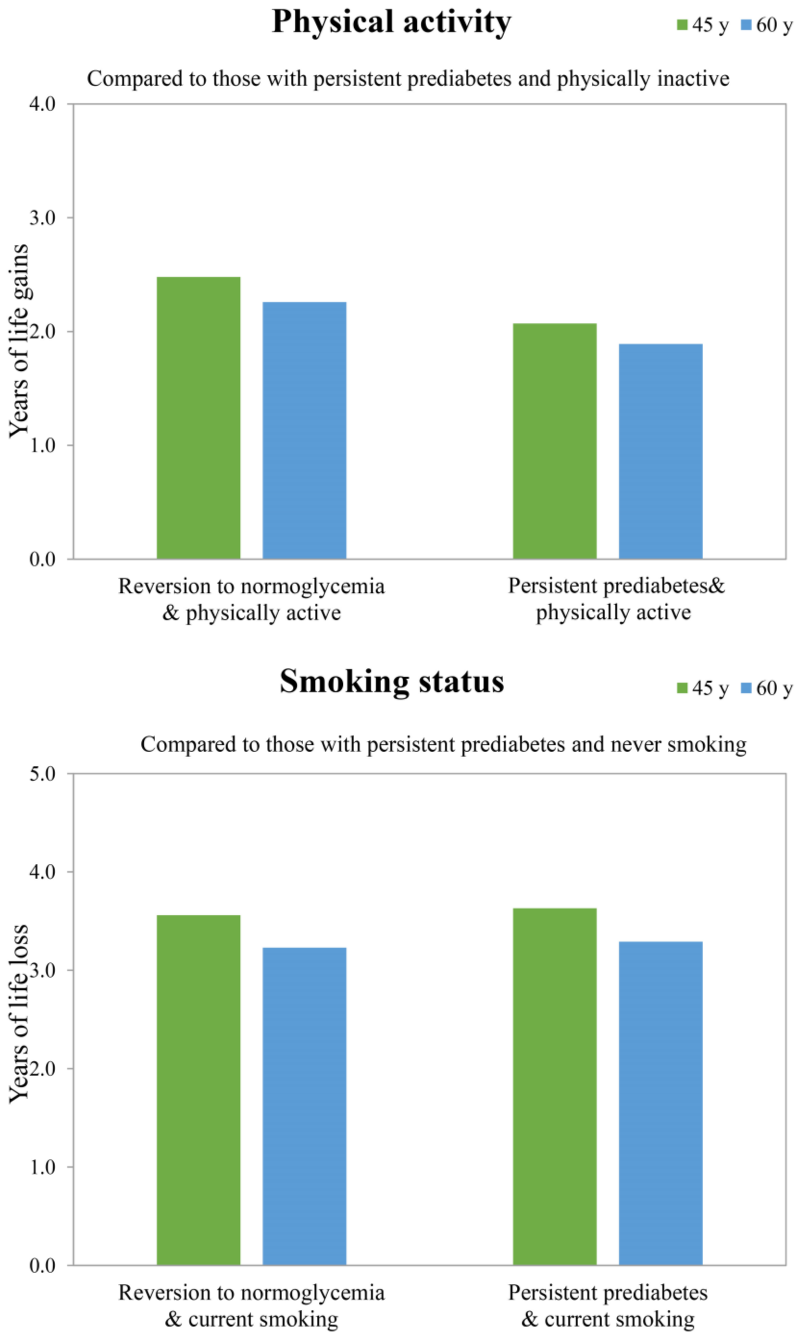

Difference of life expectancy took the persistent prediabetes and inactive physical activity or never smoking as reference group. The life expectancy was estimated by the flexible parametric survival model, which was adjusted for age, sex, educational attainment, occupation status, marriage status, smoking status, alcohol intake frequency, body mass index, physical activity, sugar-sweetened beverages, hypertension, total cholesterol.

© 2023 Cao Z et al. *JAMA Network Open*.

eFigure 4. E-Value for Associations of Progression From Prediabetes to Diabetes With All-Cause and CVD-Related Death

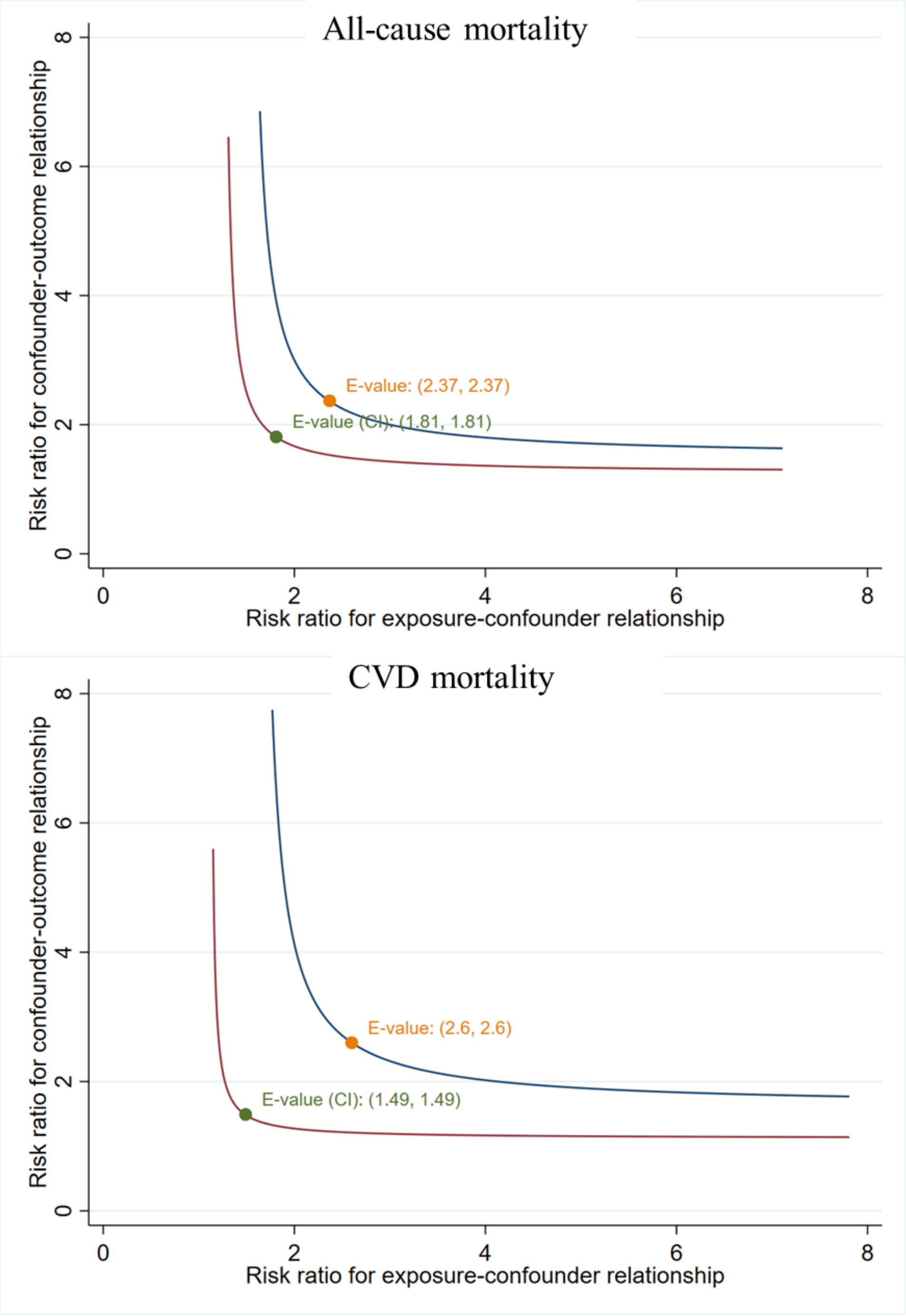

The E-value is defined as the minimum strength of association, on the risk ratio scale, that an unmeasured confounder would need to have with both the treatment and the outcome to fully explain away a specific treatment–outcome association, conditional on the measured covariates.
A large E-value implies that considerable unmeasured confounding would be needed to explain away an effect estimate. A small E-value implies little unmeasured confounding would be needed to explain away an effect estimate.

© 2023 Cao Z et al. *JAMA Network Open*.
