# Supplementary material for: Risk of Death Associated With Reversion From Prediabetes to Normoglycemia and the Role of Modifiable Risk Factors
Source: JAMA Netw Open. 2023 Mar 28;6(3):e234989. doi: 10.1001/jamanetworkopen.2023.4989 (PMC10051049; doi:10.1001/jamanetworkopen.2023.4989)
Supplement: Supplement 2. — Data Sharing Statement [file jamanetwopen-e234989-s002.pdf]

Data Sharing Statement

Cao. Risk of Death Associated With Reversion From Prediabetes to Normoglycemia and the Role of Modifiable Risk Factors. *JAMA Netw Open*. Published March 28, 2023. doi:10.1001/jamanetworkopen.2023.4989

Data
Data available: No
